# Impression Management on Instagram and Unethical Behavior: The Role of Gender and Social Media Fatigue

**DOI:** 10.3390/ijerph19169808

**Published:** 2022-08-09

**Authors:** Esraa Al-Shatti, Marc Ohana, Philippe Odou, Michel Zaitouni

**Affiliations:** 1 College of Business Administration, Gulf University for Science & Technology, West Mishref, Hawally 32093, Kuwait; 2 Sustainability Centre of Excellence, Kedge Business School Bordeaux, 33405 Talence, France; 3 UFR Sciences économiques et Gestion, University De Champagne Ardenne, 51571 Reims, France

**Keywords:** impression management concerns, wellbeing, social media fatigue, unethical behavior

## Abstract

Impression management (IM) concerns can lead to significant psychological consequences, potentially engendering unethical behavior. Therefore, adopting the stressor–strain–outcome framework, this study explores the effects of IM concerns on unethical behavior through wellbeing, and whether IM on social media (i.e., Instagram) triggers fatigue and results in unethical behavior at work. The findings of two empirical studies (n = 480 and n = 299) in different settings (Kuwait and the UK) suggest that women experience higher effects from IM concerns compared with men in Kuwait, while no gender differences are found in the UK. The results also confirm that impression management on social media platforms triggers fatigue, in turn increasing unethical behavior at work. This study contributes to the IM literature by capturing the effect of Instagram activities on workplace behavior.

## 1. Introduction

Social media is now part of everyday life. Users participate by posting images to stay in touch, connected, and updated. These platforms have played an essential role in creating and maintaining relationships [1]. For instance, the widely popular Instagram platform has changed how individuals interact with each other [2]. The proliferation of Instagram, defined as “one of the manifest transformations of everyday communication practices over the past decade” [3] (p. 358), has led to the *visual social media* distinction [4].

This phenomenon has recently motivated scholars to conduct investigations of impression management (IM) in the online world [5,6]. Indeed, due to their IM concerns, a vast number of social media users plan their posts ahead. Users tend to be more concerned about their behavior once it is more public, hence managing their impression of how their behavior and overall image appear to others [7]. As a result, impression management concerns may become a stressor leading to strain, such as social media fatigue [1,7], in turn fostering unethical behavior.

In this study, we thus examine how IM concerns in social media can lead to unethical behavior due to social media fatigue and whether gender affects this relationship. Indeed, prior research has revealed a moderating effect of gender with regard to IM and social media platforms [8], while other studies show some significant differences between genders in terms of self-presentation on social media platforms [9]. Therefore, we conducted two studies in two different settings (i.e., Kuwait and the UK) to analyze whether men and women react differently due to their IM concerns with particular regard to the Instagram platform.

## 2. Background

### 2.1. Impression Management Theory

Early conceptualization of the impression management theory can be traced to Goffman, [10] whereby individuals seek to influence others’ perceptions of themselves by regulating information in their social interactions. Goffman [10] expanded on the notion by suggesting that life is a theater, such that individuals engage in influencing impressions on a daily basis. When applied to everyday life, the essential tenet of impression management theory is that individuals enter into roleplay in different interaction modes, whether face-to-face (i.e., offline) or online [11]. Indeed, in today’s modern world, individuals and organizations increasingly operate through the online interaction mode via social networking platforms [12].

Impression management theory in the context of the online interaction mode is used as a way of influencing and controlling the image of individuals through their social media presence thanks to their profiles, activities, and the people they follow on these platforms [13]. In other words, individuals’ presence and activities on social media platforms lead to impression management concerns.

### 2.2. Impression Management Concerns and Social Media Fatigue

Social media platforms have vast audiences all around the globe, amplifying users’ concerns about their presence on these platforms, thus disclosing mainly positive behaviors and attitudes in the information they provided in their profiles. Individuals’ profiles on social media platforms are easily accessible, yielding additional information, and spreading faster than in the face-to-face or offline social interaction mode [14]. Online self-disclosure can cause negative psychological wellbeing, such as social media fatigue, related to a user’s social media presence (i.e., self-disclosure of their activities). Individuals therefore need to be cautious when using these platforms, as their presence can be tracked through self-disclosure, sharing, following, and writing comments. When the information disclosed is recognized as appropriate, it can create a good image, but when deemed inappropriate, their image deteriorates, and judgement ensues [15].

While fatigue has been studied in both interaction modes (i.e., face-to-face and online) [15], researchers tend to focus more on the online contexts, such as social media platforms [15,16]. The main consequences of social media fatigue derive from sharing information, opinions, and activities on these platforms. Numerous studies have underlined that social media users are concerned about information disclosure [15,17,18]. Indeed, the stressor–strain–outcome (SSO) theoretical framework explains the stress and fatigue associated with the use of technology, social media platforms in particular [19].

As implied, the three main components of this framework are stressor(s), strain, and outcome(s). The consequence of stressors is strain, which results in a particular outcome. In the SSO framework, stressors are defined as problematic emotional and behavioral stimuli, such as compulsive social media usage [19] leading to technostress, a consequence of the increasing use of technology, such as social media platforms [19]. Stressors resulting in social media fatigue can derive from impression management (IM) concerns. Specifically, social media platforms, such as Instagram, can trigger stress due to exposing individuals to the scrutiny of large audiences and hence society’s evaluation. As such, IM concerns can be a source of social anxiety due to the “prospect or presence of personal evaluation in real or imaged social situation” [20] (p. 642). When individuals are not perceived in the way they want to be, it stimulates a negative side of impression management, resulting in stress-related outcomes, such as burnout [21], social anxiety [22], and affecting wellbeing [23]. A recent study points out that IM concerns also cause fatigue [1].

Fatigue is a complex and universal symptom examined by occupational researchers and clinicians [24]. In the clinician’s perspective, fatigue refers to “a subjective, unpleasant feeling of tiredness that has multiple dimensions varying in duration, unpleasantness and intensity” [25]. The occupational perspective defines fatigue as “a response of vulnerable individuals to high demands or workload and inability to meet individuals’ goals” [26]. The two forms of fatigue are physical and psychological [27]. Physical fatigue refers to “functional organ failure” [28] (e.g., eye or muscle fatigue), whereas psychological fatigue refers to “a state of weariness related to reduced motivation” [24,29]. In this paper, we focus on psychological fatigue. The determinant of social media fatigue derives from psychological stress-related conditions, such as information overload and social interaction activities [30]. In particular, prior research shows that social media users with high IM concerns may experience social media fatigue [31,32]. In accordance, we posit:

**Hypothesis** **1.** *Impression management concerns are positively related with social media fatigue*.

### 2.3. Gender Differences on Impression Management Tactics

The different roles of women and men in society may affect their behavior in relation to IM concerns. Gender differences have been examined in conjunction with IM tactics, as different social roles are likely to lead to different impression management behaviors. For example, men engage more in self-promotion (the practice of enhancing their best features) and entitlement tactics (being responsible for positive incidents) than women [33,34,35]. Men also use more intimidation tactics (to have others view them as dangerous) [36], focus on status, tend to engage in more self-disclosure online [9], and exhibit unsentimental and emotionally unexpressive behavior [37], thus expressing fewer interest for IM concerns and related behaviors than women.

Conversely, women are more vulnerable to criticism, and hence use tactics such as modesty (identifying weak characteristics) [33,38], opinion conformity (agreeing with comments made by others), and compliments (flattering others) [39] compared with men. They are also more concerned about comments on their profiles, violations of online privacy, and carefully manage self-disclosure [37,40].

In summary, men generally report using some impression management tactics more, such as self-promotion, entitlement, intimidation, boasting, and blurring, while women engage more in direct impression management tactics, such as modesty, opinion conformity, and compliments. As such, consequences of impression management concerns are higher for women than men because their role as women requires more sentimental and emotional behaviors. Women also tend to receive more criticism about their profiles than men; thus, are subject to further stress, higher strain, and social media fatigue. Hence:

**Hypothesis** **2.** *Gender moderates the relationship between impression management and social media fatigue such that the relationship will be stronger for women than men*.

### 2.4. Fatigue and Unethical Behavior

Social media makes the world smaller, allowing individuals to stay up to date with information, news, fashion, politics, and education. However, there is also a dark side to social media that may trigger unethical behavior defined as actions that may harm individuals or organizations and are “illegal or morally unacceptable by society” [41] (p. 367). Scholars argue that social media fatigue has a negative effect at the individual level [16,42] both mentally and physiologically, whereby individuals are likely to develop unethical behaviors [43]. Users may also experience a decline in life satisfaction due to the lack of motivation ensuing from social media fatigue [44]. Fatigue causes attention and working memory impairment, lack of cognitive flexibility and decision making and, in turn, unethical behaviors [45]. In addition, the features of social media platforms may enable mechanisms that facilitate moral disengagement and thus the enactment of unethical behaviors [46]. Moreover, fatigue can cause sleep problems, considered a potential antecedent of unethical behavior [47].

Generally, fatigued individuals tend to have a lower self-regulatory capacity that enhances their engagement in unethical behavior [48]. Neuropsychology and physiology are the main underpinning mechanisms of the self-regulatory theory. According to the self-regulatory fatigue literature, physical fatigue refers to ‘a “catastrophic” failure of homeostasis due to a depletion of biological substrates, a buildup of toxins, or both have persisted throughout time’ [49], while cognitive fatigue refers to ‘a psychobiological state caused by prolonged period of demanding cognitive activity’ [50,51,52]. Getting fatigue due to excessive usage of social media platforms (i.e., being constantly on the screen) creates a cognitive fatigue (i.e., neuropsychology). Therefore, this research paper focuses on the neuropsychological fatigue rather than the physical.

Several models can explain the relationship between fatigue and unethical behavior. According to the self-regulation theory [53,54], when an individual’s self-control is depleted (i.e., ego depletion), their cognitive resources are strained, thereby hindering the ability to self-regulate their behaviors. In the same way that individuals use self-control to manage their weight, they can use it to avoid engaging in unethical behavior (e.g., submitting an exaggerated expense report in the workplace). Specifically, the more stressors an individual experiences, the harder it is to exert self-control. In this vein, studies highlight that self-control depletion tends to foster cheating [55]. Individuals with high fatigue due to depleted self-regulatory resources engage in unethical behavior as a coping mechanism on the assumption that these behaviors will benefit the organization or self [48].

The motivational control theory of cognitive fatigue, which indicates that failure of energy affects changing in behavior and control of goals can also explain the relationship between fatigue and unethical behavior. According to this view, fatigue is an emotion that interjects current behavior and creates a conflict between duties and desires [56]. This motivated switching between the job and the leisure allows people not only to mentally engage in the task, but also to disengage from it and look for activities that are more fun. Consequently, individuals with high cognitive fatigue will disengage from their jobs in order to satisfy their leisure, thus translating into unethical behavior. This shows how cognitive fatigue that is resulted from building impressions and being active on social media can cause individuals to be less motivated to control their goals and therefore to practice unethical behavior [57]. In other words, the brain guides people toward enjoyable activities more than activities they ought to be focusing on because of particular obligations [58]. Therefore, social media usage results in fatigue that may lead to unethical behavior, hence:

**Hypothesis** **3.** *Social media fatigue is positively related to unethical behavior*.

## 3. Method

**Figure 1:**
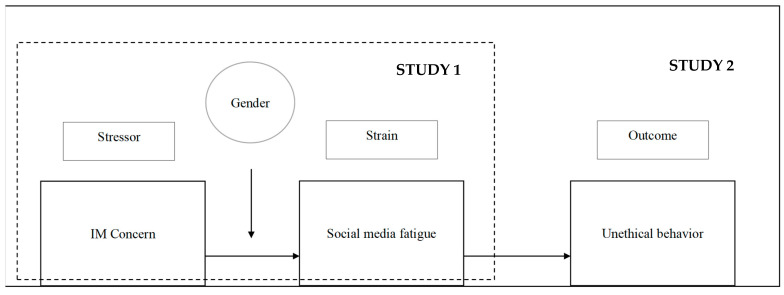
Research model.

To test our hypotheses, we conducted two studies, as detailed next. Figure 1 presents our research model.

### 3.1. Study 1

The first study aimed at testing the moderating role of gender in the relationship between IM concerns and social media fatigue.

#### 3.1.1. Sample

Participants were recruited in September 2021 from Kuwait through a social media influencer with 720 k followers, who announced the study and posted a link on his Instagram story with the “swipe up option” were people can access the questionnaire to participate in the study. As the original items in the questionnaire were in English, a back-translation method was used to convert the items into Arabic by a validated translation company in Kuwait and given to two other bilingual individuals to translate them from Arabic to English. We then checked the original scale to make sure it delivered the same meaning. Participants were assured anonymity and participation was voluntary; they were also free to withdraw at any time. No personal questions were asked. Any missing responses of the main variables were excluded and the inclusion criteria was age related, resulting in a final sample of 480 participants of whom 60% were female. The age of participants was from 18 to 55+, asked on a category manner (1 = “18–25”, 2 = “26–35”, 3 = “36–45”, 4 = “45–55”, and 5 = “55+”). The minimum age was 18 and only 4 participants were aged more than 55. The average time participants spent on social media was almost 3 h per day.

#### 3.1.2. Measures

*Impression management concerns* were measured using four items [1] on a 7-point scale (1 = strongly disagree to 7 = strongly agree). A sample item is: “I am concerned about saying socially inappropriate things on social media platforms (i.e., Instagram)”. Cronbach’s alpha for this measure is 0.83.

*Social media fatigue* was measured using four items adapted from Dhir et al., [15], with responses made on a 7-point scale (1 = strongly disagree to 7 = strongly agree). A sample item is: “I find it difficult to relax after continually using social media platforms (i.e., Instagram)”. Cronbach’s alpha for this measure is 0.85.

*Gender* is a categorical variable, coded 1 for male and 2 for female.

#### 3.1.3. Results and Data Analysis

Table 1 represents the mean, standard deviation, and intercorrelations of the variables under study. All the measures are found reliable with Cronbach’s alpha above 0.70. We used the SPSS statistical software (version 25.0). The results indicate that IM concerns have a significant positive relation with social media fatigue (*r* = 0.488, *p <* 0.01) and a significantly negative relation with gender (*r* = −0.29, *p* < 0.01). Social media fatigue has a positive relation with gender (*r* = 0.21, *p <* 0.01). We also tested this relation with a hierarchical regression analysis. That result indicates that IM concerns (*β* = 1.187, *p <* 0.01) are significantly related with social media fatigue, as shown in Table 2. Thus, Hypothesis 1 is supported.

**Figure 2:**
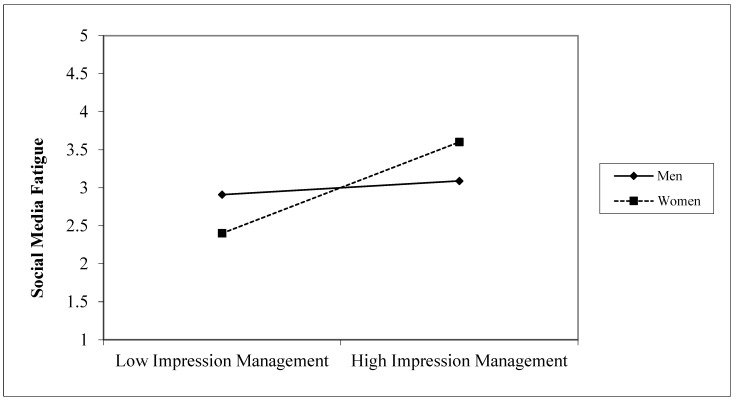
Interaction between social media fatigue and IM concerns on gender.

To test for moderation, we used PROCESS (model 1), an SPSS macro developed by Hayes [54], with 5000 bootstrapped samples to assess the relationships. We examined whether the effect of X (IM concerns) on Y (social media fatigue) is moderated by M (gender). The results presented in Table 2 show that the interaction term is significant, as 0 does not fall within the confidence interval. In addition, the conditional effect shows that for males the model is highly significant (*effect* = 0.86, *SE* = 0.122, *t* = 7.07, the confidence interval does not contain zero, *p <* 0.01), indicating a moderating relationship with IM concerns and social media fatigue. Furthermore, the relationship for males is weaker than for females (*effect* = 1.377, *SE* = 0.09, *t* = 14.6, the confidence interval does not contain zero, *p <* 0.01). As Figure 2 shows, the effect is consistent with our expectations, thus providing support for Hypothesis 2.

### 3.2. Study 2

#### 3.2.1. Sample

In this second study, the data were collected in February 2022. Participants were recruited from the UK through the Prolific online survey platform, which provides adequate data quality [59]. The purpose of the study was explained at the beginning of the survey. It targeted people with a high interest in social media in order to focus on social media fatigue and working people (full time, at least 31 h per week, and not student status) to test for unethical behavior at work. A total of 299 individuals participated, of whom 51% were male and 49% were female. Participants had between a minimum of one year and a maximum of 50 years’ experience in their organizations and an average age of 35 years (minimum of 19 and maximum of 59).

#### 3.2.2. Measures

We measured IM concerns (Cronbach’s alpha = 0.88) and social media fatigue (Cronbach’s alpha = 0.90) using the same scales as in Study 1.

*Unethical behavior* was measured with five items adapted from Zuber and Kaptein [60] on a 5-point scale (1 = strongly disagree to 5 = strongly agree). A sample item is: “I discriminate against employees (on the basis of age, race, gender, religious belief, sexual orientation, etc.)”. Cronbach’s alpha for this measure is 0.94.

*Gender* is a categorical variable tested as moderator coded 1 for female and 2 for male.

#### 3.2.3. Results and Data Analysis

Table 3 shows the mean, standard deviation, and intercorrelations of the variables for Study 2. We first tested the link between IM concerns and unethical behavior with the mediation effect of social media fatigue using SPSS Process, Model 4. Table 4 presents the mediation results. The results indicate a significant effect of IM concerns on social media fatigue (*β* = 0.11, *p < 0*.05). The results also show a statistically significant effect of social media fatigue on unethical behaviors (*β =* 0.06, *p* < 0.05). The effect of IM concerns on unethical behavior in the presence of SM fatigue is also significant (*β* = −0.04, *p* < 0.05), while the indirect effect is 0.0063, 95% CI = [0.001;0.01], hence confirming the existence of mediation. It thus confirms Hypotheses 1 and 3.

Table 5 presents the results of the moderated mediation analyses (PROCESS, Model 7) [61] to test Hypothesis 2. The upper part of Table 5 shows there is no interaction between IM concerns and gender (*β* = 0.012, *p* = 0.90). We probed the conditional indirect effect of IM concerns on unethical behavior through social media fatigue at three values of gender (i.e., at the mean and at one standard deviation below and above the mean). The results, shown at the bottom of Table 5, indicate that the conditional indirect effect is non-significant for both genders. The index of moderated mediation [62] is equal to 0.0007 and its confidence interval ([−0.012;0.013]) includes zero. Together, the results do not support Hypothesis 2 or any moderated mediation relationship.

## 4. Discussion

This study examines the effect of IM concerns on unethical behavior by introducing social media fatigue as the mediator and gender as the moderator of this relationship. The results show that IM concerns are sensitive to gender in certain circumstances, as gender reactions to high self-exposure on social media platforms (i.e., Instagram) may increase the level of social media fatigue. The results also show that for individuals with high fatigue, the effect of IM concerns on unethical behavior is more salient. Our findings yield several theoretical and managerial implications as discussed next.

### 4.1. Theoretical Implications

This research provides several contributions to the literature. First, our study enhances current understanding of the consequences of IM concerns on unethical behavior in response to several calls for more research on this subject [63,64]. Although prior studies have examined the negative effect of IM on unethical behavior, our study goes further by stressing the importance of the online context. The ubiquitous use of social media influences the day-to-day activities of individuals, including when they are at work. Indeed, our results show that social media fatigue is carried from the personal to the work level. Despite ample studies examining this relationship in the workplace, to our best knowledge, no studies examine how matters that occur outside of work penetrate the workplace context. Thus, our first contribution is the analysis of IM deriving from social network use in private life as Sun et al., [65] and Oh and LaRose [7] suggest, highlighting the need to investigate personal IM concerns around social networks to understand unethical work behavior.

Second, our two studies confirm an underlying wellbeing mechanism to explain the relationship between IM concerns and unethical behavior [66]. Although prior studies consider the direct relationship between IM and unethical behavior [67], our contribution focuses on the underlying psychological mechanisms in the IM literature to analyze the mediating mechanism in terms of fatigue, health, and wellbeing. Following research that links fatigue and unethical behavior [68], we contribute to the IM literature by integrating the self-regulatory theory, explaining that when individuals feel drained, they are more likely to behave unethically as their ability to self-control reduces. In particular, individuals need to maintain their ability to self-control to prevent engaging in unethical behavior at the workplace. Therefore, our study contributes by integrating the self-regulatory theory in the IM literature to capture how Instagram activities affect workplace behaviors.

Finally, our research contributes to the IM literature by examining the complex effects of IM on employees’ unethical behavior. Specifically, we introduce a moderator, namely gender, to better understand this complexity. Surprisingly, we found different results for our two study samples: in the UK sample, gender has no moderating role, whereas in Kuwait, women show a stronger effect of IM on social media fatigue. The differing results can be explained by the societal specificities in these two countries. The first study in Kuwait, known for its more conservative society, shows that men are more willing to ignore managing their IM concerns on social media platforms and avoid social media fatigue. Conversely, women tend to be more sensitive to their IM concerns, because they worry more about society’s judgments. On the other hand, our second study conducted in the UK, which has a more open society, shows no differences in terms of gender. In particular, men and women in the UK have the same concerns when managing their social media impressions.

### 4.2. Limitations and Future Directions

Despite these significant contributions, our study is not free from limitations. First, our study only focuses on IM concerns, while ignoring other stressors that cause social media fatigue. Second, we focus on IM concerns and unethical behavior without considering other behavioral outcomes that may arise from fatigue and strain. However, we hope future studies will address these relevant research avenues.

## 5. Conclusions

Our study shows the important role of IM concerns in the online context using social media fatigue as the mediator and gender as the moderator to describe the IM–unethical behavior relationship. Based on insights from the self-regulatory theory, the SSO framework, and impression management views, this study advances current research by investigating the impact of IM concerns on unethical behavior. Finally, our study suggests that gender only has a moderating effect in conservative countries and no effect in relation to IM concerns on social media platforms in more open societies.

## Figures and Tables

**Table 1:** Study 1: mean, standard deviation, and intercorrelations.

	Mean	SD	1	2	3	4
IM concerns	2.55	0.89	1	0.488 **	−0.290 **	0.025
Social media fatigue	3.04	1.76	0.488 **	1	0.213 **	−0.07
Gender	1.6	0.49	−0.290 **	0.213 **	1	−0.072
Time spent on Instagram	2.86	1.159	0.025	−0.07	−0.072	1

** Correlation significant at the 0.01 level (2-tailed).

**Table 2:** Moderating role of gender (M) on the relationship between IM concerns (X) and social media fatigue (Y).

Model Summary			95% CL				
		*β*	*SE*	*t*	*p*	*LL*	*UL*
	Constant	3.1	0.066	46.79	<0.001	2.97	3.23
	IM concerns (X)	1.18	0.074	15.63	<0.001	1.02	1.31
	Gender (M)	1.31	0.136	9.66	<0.001	1.05	1.58
	Int_term (XM)	0.0512	0.154	3.31	0.001	0.2	0.81
*Conditional effect of X on Y at values of the moderator:*							
Gender (M)	*Effect*	*SE*	*t*	*p*	*LLCI*	*ULCI*	
Male	0.865	0.122	7.073	<0.001	0.062	1.105	
Female	1.377	0.094	14.608	<0.001	1.19	1.562	

Note: CL—confidence interval; LL—lower limit; UL—upper limit, X—independent variable, Y—dependent variable; M—moderator; Int_term—IM concerns X gender.

**Table 3:** Study 2: mean, standard deviation, and intercorrelations.

	Mean	SD	1	2	3	4
IM concerns	3.19	1.09	1	0.135 *	−0.09	−0.05
Social media fatigue	2.14	0.9	0.135 *	1	0.11	−0.07
Unethical behavior	1.13	0.4	−0.09	0.11	1	0.049
Gender	1.51	0.5	−0.05	−0.07	0.049	1

* Correlation significant at the 0.05 level (2-tailed).

**Table 4:** Regression results for simple mediation.

	*Coeff.*	*t*	*p*		
IM concerns regressed on social media fatigue	0.11	2.35	<0.05		
IM concerns regressed on unethical behavior	−0.041	−1.96	<0.1		
Social media fatigue regressed on unethical behavior	0.056	2.17	<0.05		
	Bias-corrected bootstrap confidence interval based on 10,000 bootstrap samples				
			95% confidence interval limits		
	*Effect*	*SE*	*Lower*	*Upper*	
Indirect effect	0.0063	0.0046	0.0003	0.0177	

**Table 5:** Results of the moderated mediation analysis.

Predictor	*β*	*SE*	*t*	
DV: Social media fatigue				
Constant	2.14	0.052	41.15 ***	
IM concerns	0.1	0.047	2.28 *	
Gender	−0.11	0.103	−1.08	
IM concerns x Gender	0.012	0.094	0.129	
DV: Unethical behavior				
Constant	1.01	0.059	16.96 ***	
IM concerns	−0.042	0.021	−1.96	
Social media fatigue	0.055	0.026	2.18 *	
Conditional indirect bootstrap estimates for social media fatigue				
Gender	*Boot indirect effect*	*Boot SE*	*LL CI*	*UL CI*
Female	0.0057	0.0056	−0.0012	0.0200
Male	0.0059	0.0052	−0.0016	0.0181

*** *p* < 0.001; * *p* < 0.05.

## Data Availability

Data can be obtained from the contact author.
